# Clinical relevance of systemic monocytic-MDSCs in patients with metastatic breast cancer

**DOI:** 10.1007/s00262-019-02472-z

**Published:** 2020-01-10

**Authors:** Caroline Bergenfelz, Anna Roxå, Meliha Mehmeti, Karin Leandersson, Anna-Maria Larsson

**Affiliations:** 1 Department of Translational Medicine, Cancer Immunology, Lund University, 21428 Malmö, Sweden; 2 Department of Translational Medicine, Experimental Infection Medicine, Lund University, Inga Marie Nilssons gata 53, 21428 Malmö, Sweden; 3 Department of Clinical Sciences Lund, Division of Oncology and Pathology, Lund University, Lund, Sweden; 4 Department of Hematology, Oncology and Radiation Physics, Skåne University Hospital, Lund, Sweden

**Keywords:** Breast cancer, Metastasis, Mo-MDSCs, Survival, Estrogen receptor

## Abstract

The overall aim of this prospective study was to delineate the role of monocytic myeloid-derived suppressor cells (Mo-MDSCs) in patients with metastatic breast cancer (MBC). MDSCs are a heterogeneous group of immunosuppressive cells often enriched in different malignancies which hold prognostic and predictive value for clinical outcomes. Here, we assessed the clinical significance of Mo-MDSCs in 54 patients with de novo or distant recurrent MBC. We show that high levels of Mo-MDSCs significantly correlated with de novo MBC (metastatic disease at initial diagnosis), estrogen receptor (ER) negativity, and liver- and bone metastasis. A trend towards an association between high levels of Mo-MDSCs and survival (*P* = 0.053) was also found in patients with distant recurrent ER-positive MBC. We therefore propose that an increased population of Mo-MDSCs may be related to the metastatic or immunoregulatory switch associated with transition to a more systemic disease. Our data imply that high levels of systemic Mo-MDSCs represent patients with more aggressive disease and worse outcome.

**Electronic supplementary material:**

The online version of this article (10.1007/s00262-019-02472-z) contains supplementary material, which is available to authorized users.

## Introduction

Breast cancer is the most common form of malignancy in women [1] and although survival has improved, due to enhanced diagnostics and adjuvant therapies, breast cancer can recur after many years [2, 3]. When there is progression to metastatic breast cancer (MBC), there is in most cases no curable treatment and the 5-year survival rate is merely 20–30% [2, 4].

Over the past decades, the role of the immune system in cancer development and progression has gained increased attention. Although the exact mechanisms are still being investigated, it is known that some immune cells are able to recognize and eliminate tumor cells, a mechanism called immunosurveillance [5, 6]. In order for cancer to develop and progress, malignant cells must escape this surveillance and hijack normal physiological processes typically involved in tissue remodeling, angiogenesis, and cell regeneration [6-8]. One mechanism by which tumors can elude immunosurveillance is through myeloid-derived suppressor cells (MDSCs). MDSCs are a heterogeneous group of highly immunosuppressive cells that accumulate during severe pathological conditions such as sepsis, trauma and cancer [9-11]. Physiologically, they function to dampen excessive immune responses and limit or promote repair of tissue damage [9, 10]. In cancer, however, the net result would be tumor persistence and progression due to the production of immunosuppressive and pro-angiogenic cytokines and inhibition of anti-tumor T cell responses [10, 12]. The role of MDSCs in breast cancer patients remains relatively unexplored.

Depending on cell surface antigen expression, MDSCs have historically been roughly divided into two groups; granulocytic-MDSCs (G-MDSCs; CD11b^+^CD15^+^CD33^+^Lin^−^HLA-DR^low/−^) and monocytic-MDSCs (Mo-MDSCs; CD11b^+^CD14^+^CD33^+^HLA-DR^low/−^Co-receptor^low/−^) [12]. Generally, MDSCs correlate with tumor progression, angiogenesis and poor prognosis in different cancer forms [13-15]. Accordingly, in breast cancer patients, MDSCs are enriched in the peripheral blood and has been proposed to correlate with clinical stage and metastatic burden [16-19]. In patients with stage IV disease, higher levels of MDSCs have also been associated with reduced overall survival [19, 20]. However, the surface definition of MDSCs has varied substantially between studies and many do not distinguish between G-MDSCs and Mo-MDSCs. A recent study proposes that G-MDSCs (CD11b^+^CD33^+^HLA-DR^−^CD15^+^ cells) may correlate with triple negative breast cancer and inversely with complete pathologic response after neo-adjuvant chemotherapy [21]. Historically, Mo-MDSCs have been less studied. Enrichment of circulating Mo-MDSCs has previously been identified in patients with melanoma [22, 23], prostate cancer [24], glioblastoma [25], non-Hodgkin lymphoma [26], and bladder cancer [27]. Depending on the type of malignancy, amongst other factors, the presence of Mo-MDSCs correlated with more active [22] or aggressive disease [26] as well as increased tumor size and grade [27]. Whether Mo-MDSCs may provide prognostic or predictive value in patients with breast cancer is currently unknown.

We were recently first to show that Mo-MDSCs are enriched in the peripheral blood of breast cancer patients, primarily in patients with metastatic disease [28]. Here, we aim to further delineate the role of Mo-MDSCs in patients with MBC. By analyzing the frequency of CD14^+^HLA-DR^low/−^Co-receptor^low/−^ Mo-MDSCs in the peripheral blood of patients with MBC we show that Mo-MDSCs significantly correlate to estrogen receptor (ER)-negative disease, liver- and bone metastases, and de novo MBC (metastatic disease at initial diagnosis). Furthermore, in patients with distant recurrent MBC, high levels of Mo-MDSCs were associated with more circulating tumor cells (CTCs), more metastatic sites and disease progression at 3 month’s radiology assessment. In patients with ER-positive distant recurrent MBC, there was a trend towards an association between high levels of Mo-MDSCs and worse overall survival (*P* = 0.053). Altogether this indicates that Mo-MDSCs may be a potential biomarker related to a more aggressive disease and worse outcome in patients with MBC.

## Materials and methods

### Patients and study design

54 patients, with newly diagnosed MBC, scheduled for first line systemic treatment at Skåne University Hospital in Lund, were enrolled between the years 2011 and 2016. This is a sub-study of the prospective observational CTC-MBC study (ClinicalTrials.gov NCT01322893) and clinical information has previously been published [29]. All blood samples were collected before starting systemic therapy for metastatic disease. 13 patients were, however, on endocrine therapy for early breast cancer at the time of MBC diagnosis (see Supplementary Table 1, where previous and current therapy at MBC diagnosis are indicated). The median follow-up time was 27 months for patients alive at the last medical visit before follow-up cut-off date 2017-05-31. Inclusion criteria were: MBC diagnosis, age ≥ 18 years, Eastern Cooperative Oncology Group (ECOG) performance status 0–2, and predicted life expectancy of > 2 months. Exclusion criteria were: prior systemic therapy for metastatic disease, inability to understand the study information, and other malignant disease in the preceding 5 years. Structured clinical and radiological evaluations were performed every 3 months. Progression was defined as progressive disease (PD), whereas non-progression was defined as stable disease (SD) or partial or complete regression (PR and CR), using modified RECIST 1.1 criteria [30]. Patient, tumor, and treatment data were prospectively collected in structured clinical report forms (CRFs). 22 anonymized healthy blood donors were used as controls.

### Flow cytometric analysis of Mo-MDSCs

Peripheral blood was collected in EDTA-coated tubes at baseline, before starting systemic therapy, and analyzed within 24 h as previously described [28]. Briefly, the peripheral blood was diluted in phosphate-buffered saline (PBS; EDTA/sucrose) and overlaid on Ficoll-Paque Plus (cat no. 17-1440-03, GE Healthcare). The peripheral blood mononuclear cells (PBMCs) were collected, washed once in FACS buffer (PBS; BSA/EDTA), and immediately stained for flow cytometry. Antibodies used were as follows: CD14 clone M5E2 (1:10; cat. no 555397), HLA-DR clone G46-6 (1:50; cat. no. 347403), CD80 clone L307.4 (1:15; cat no. 557227), CD86 clone IT2.2 (1:15; cat no. 555665), CD33 clone WM53 (1:10; cat. no 551378), all from BD Biosciences. Analyses were performed using a FACS Calibur (BD Biosciences) and on gated viable (7AAD-negative) PBMCs. Mo-MDSCs were defined as CD14^+^HLA-DR^low/−^ cells, where these cells were also deemed CD33^+^ and with low expression of the co-receptors CD86 and CD80 compared to CD14^+^HLA-DR^+/high^ cells as well as being immunosuppressive, as demonstrated by their suppression of T cell proliferation in an ex vivo assay (for full characterization see Bergenfelz et al. [28]). The immunosuppressive profile of Mo-MDSCs from MBC patients was confirmed in a previous study, where flow cytometric analyses of immune cell populations from the first 23 patients included here also have been published [28].

### Detection of CTCs

Blood samples for detection of circulating tumor cells (CTCs) were collected in 10 mL CellSave Preservation tubes (Menarini Silicon Biosystem, cat. no 7900005), stored between 15 and 30° C and processed within 96 h of collection. CTC enumeration was performed using the CellSearch system (Menarini Silicon Biosystems) as has been described in detail previously [29, 31, 32]. The established cut-off at 5 CTCs was used for defining low levels (< 5 CTCs) versus high levels (≥ 5 CTCs) [29, 31].

### Statistical analyses

Comparison of Mo-MDSC levels in patients and healthy controls (HC) was made with non-parametric Mann–Whitney Wilcoxon test (for comparison between two groups) and Kruskal Wallis and Dunn’s multiple comparison test (for comparison between more than two groups). Categorical patient and tumor characteristics in relation to Mo-MDSC levels were compared using Pearson’s chi-squared test and ordinal data were compared using Pearson’s chi-squared test for trend. If expected counts were lower than five in one or more of the cells, Fisher’s exact test was used. Survival was calculated and illustrated by the log-rank test and Kaplan–Meier curves. Statistical analysis was performed with IBM SPSS Statistics (version 24.0, IBM) and graphs were made with GraphPad Prism (version 8, GraphPad Software).

## Results

### Patient characteristics

54 patients with newly diagnosed MBC were included in this prospective study (Table 1). The median age at MBC diagnosis was 65 years (range 40–84). 36 patients (67%) had ER-positive primary tumors, 12 (22%) had ER-negative primary tumors, and seven (13%) were HER2-positive (five ER-positive and two ER-negative). 21 patients (39%) had three or more metastatic sites and 34 patients (63%) had visceral metastasis. Bone metastases were most prevalent (42 patients; 78%), followed by lymph node and lung metastases (23 patients; 43% and 21 patients; 39%, respectively). 16 patients were diagnosed with liver metastasis (30%). 12 patients (22%) had de novo MBC at initial breast cancer diagnosis, while 42 patients (78%) in the studied group were diagnosed with distant recurrence. The median metastasis-free interval (MFI) for patients with distant recurrent MBC was 6.4 years (range 1.0–27.2). Patient characteristics are summarized in Table 1.

**Table 1:** Clinicopathological variables in all patients, stratified by levels of Mo-MDSCs

Clinicopathological variable	All patients *n* = 54	Normal Mo-MDSCs *n* = 28 (%)	High Mo-MDSCs *n* = 26 (%)	*P* value
Age (years)				
< 65	27	13 (46)	14 (54)	0.59^a^
≥ 65	27	15 (54)	12 (46)	
Baseline^c^ ECOG^d^				
0	35	20 (71)	15 (60)	0.71^b^
1	6	3 (11)	3 (12)	
2	12	5 (18)	7 (28)	
Unknown	1	0	1	
Tumor type				
Ductal	40	22 (82)	18 (72)	0.45^b^
Lobular	9	3 (11)	6 (24)	
Other	3	2 (7)	1 (4)	
Unknown	2	1	1	
PT NHG				
I	4	3 (13)	1 (8)	0.88^b^
II	23	14 (58)	9 (69)	
III	10	7 (29)	3 (23)	
Unknown	17	4	13	
PT tumor size				
T1	20	13 (50)	7 (32)	0.38^b^
T2	14	8 (31)	6 (27)	
T3	7	2 (8)	5 (23)	
T4	7	3 (11)	4 (18)	
Unknown	6	2	4	
PT node status				
Negative	15	11 (42)	4 (20)	0.11^a^
Positive	31	15 (58)	16 (80)	
Unknown	8	2	6	
PT hormone receptor status				
ER-negative	12	3 (12)	9 (41)	**0.02**^**a**^
ER-positive	36	23 (88)	13 (59)	
Unknown	6	2	4	
PR-negative	19	8 (31)	11 (52)	0.13^a^
PR-positive	28	18 (69)	10 (48)	
Unknown	7	2	5	
PT HER2 status				
HER2-negative	32	18 (86)	14 (78)	0.68^b^
HER2-positive	7	3 (14)	4 (22)	
Unknown	15	7	8	
MET hormone receptor status				
ER-negative	7	2 (8)	5 (23)	0.23^b^
ER-positive	40	23 (92)	17 (77)	
Unknown	7	3	4	
PR-negative	27	13 (54)	14 (64)	0.52^a^
PR-positive	19	11 (46)	8 (36)	
Unknown	8	4	4	
MET HER2 status				
HER2-negative	39	19 (83)	20 (95)	0.35^b^
HER2-positive	5	4 (17)	1 (5)	
Unknown	10	5	5	
Metastatic sites, *n*				
< 3	33	20 (71)	13 (50)	0.11^a^
≥ 3	21	8 (29)	13 (50)	
Metastatic sites, localization				
Lymph node				
Negative	31	18 (64)	13 (50)	0.29^a^
Positive	23	10 (36)	13 (50)	
Lung				
Negative	33	16 (57)	17 (65)	0.54^a^
Positive	21	12 (43)	9 (35)	
Liver				
Negative	38	23 (82)	15 (58)	** < 0.05**^**a**^
Positive	16	5 (18)	11 (42)	
Bone				
Negative	12	10 (36)	2 (8)	**0.01**^**a**^
Positive	42	18 (64)	24 (92)	
Visceral^e^				
Non-visceral	20	11 (39)	9 (35)	0.72^a^
Visceral	34	17 (61)	17 (65)	
Bone only				
Not bone-only	43	22 (79)	21 (81)	0.84^a^
Bone-only	11	6 (21)	5 (19)	
Progression at 3 mo evaluation				
Non-progression	39	23 (92)	16 (73)	0.12^b^
Progression	8	2 (8)	6 (27)	
Unknown	7	3	4	
Type of MBC				
De novo^f^	12	2 (7)	10 (38)	**0.006**^**a**^
Distant recurrent^g^	42	26 (93)	16 (62)	
CTC at baseline^c^				
< 5	25	16 (59)	9 (35)	0.07^a^
≥ 5	28	11 (41)	17 (65)	
Unknown	1	1	0	

Number of patients and percentage (%) distribution of patients indicated for each variable

*Mo-MDSCs* monocytic myeloid-derived suppressor cells, *MBC* metastatic breast cancer, *ECOG* Eastern Cooperative Oncology Group, *PT* primary tumor, *NHG* Nottingham histological grade, *ER* estrogen receptor, *PR* progesterone receptor, *HER2* human epidermal growth factor receptor 2, *MET* metastasis, *CTC* circulating tumor cells

^a^Statistics by Pearson’s chi-squared test, significance level defined as *P* < 0.05 (bold)

^b^Statistics by Fisher’s exact test. Used when presence of expected values less than 5. Significance level defined as *P* < 0.05 (bold)

^c^Baseline defined as a time point before starting first line systemic MBC treatment

^d^ECOG denotes the performance status used in clinical practice in Sweden

^e^Visceral metastasis defined as lung, liver, brain, peritoneal, and/or pleural involvement

^f^De novo MBC defined as MBC at initial breast cancer diagnosis

^g^Distant recurrent MBC defined as MBC diagnosis after >0 years after primary diagnosis

### Mo-MDSCs are enriched in the peripheral blood of a subpopulation of MBC patients

**Fig. 1:**
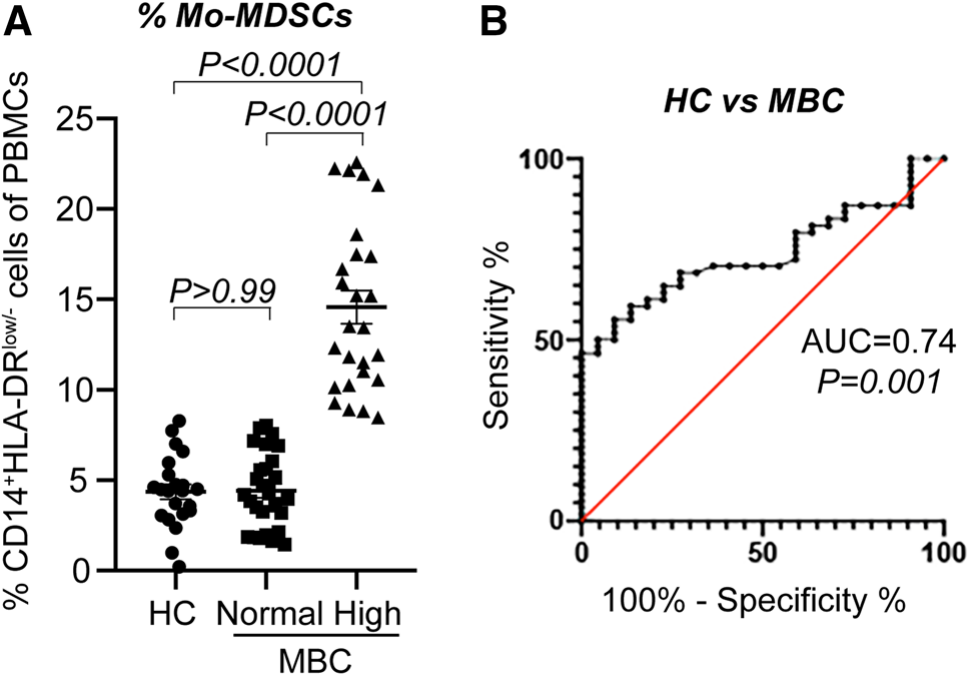
Mo-MDSCs are enriched in the peripheral blood of MBC patients. Peripheral blood was collected from 54 patients with metastatic breast cancer (MBC) and 22 healthy controls (HC). Percentage of CD14^+^HLA-DR^low/−^ Mo-MDSCs was assessed by flow cytometry. **a** Median percentage of Mo-MDSC in HC and MBC patients with normal (*n* = 28) and high (*n* = 26) levels of Mo-MDSCs. Cut-off level between normal and high levels of Mo-MDSCs was set to the highest value of Mo-MDSCs in HCs (8.29% of PBMCs), as described in [28]. Error bars; SEM. Exact *P* values, by Kruskal Wallis with Dunn’s multiple comparison test, are indicated. **b** Receiver operating characteristic (ROC) curve of percentage Mo-MDSCs in healthy controls (HC, *n* = 22) and MBC patients (*n* = 54). Area under the curve (AUC) and significance are indicated

Peripheral blood was collected from all patients at baseline, before starting systemic treatment for MBC. Circulating Mo-MDSCs (CD14^+^HLA-DR^low/−^Co-receptor^low/−^ cells) in the PBMC fraction were analyzed by flow cytometry. Gating strategies and representative mean fluorescence intensity (MFI) for co-receptors are depicted in Supplementary Fig. 1. The suppressive activity of the monocytes (suppression of T lymphocytes and increased release of IL-10) has previously been characterized in a study where we analyzed Mo-MDSCs from the first 23 patients enrolled [28]. In accordance with this study, Mo-MDSCs were significantly enriched in the peripheral blood of a subpopulation of MBC patients (Fig. 1a and Supplementary Fig. 1a). Based on a previously defined cut-off (highest Mo-MDSC value received from healthy controls [28]; 8.29% of PBMCs), the MBC patients were stratified into groups with “normal” or “high” Mo-MDSC levels. 28 patients displayed normal levels of Mo-MDSCs (range 1.45–8.03% of PBMCs) and 26 patients had high levels (range 8.47–22.57% of PBMCs, Fig. 1a). No significant difference with regards to levels of Mo-MDSCs was observed between patients on endocrine therapy or not at the time of MBC diagnosis nor for patients that previously had received adjuvant chemo- or endocrine therapy (Supplementary Table 1).

In order to assess the performance of Mo-MDSCs as a biomarker to distinguish healthy control and MBC, we used receiver operating characteristic (ROC) analyses. The frequency of Mo-MDSCs achieved an area under the curve (AUC) value of 0.74 (95% CI 0.628–0.845, *P* = 0.0013) to distinguish MBC patients from healthy controls (Fig. 1b). This indicates that the levels of Mo-MDSCs may function as a biomarker in MBC.

### Mo-MDSCs correlate with ER-negativity and liver or bone metastases

**Fig. 2:**
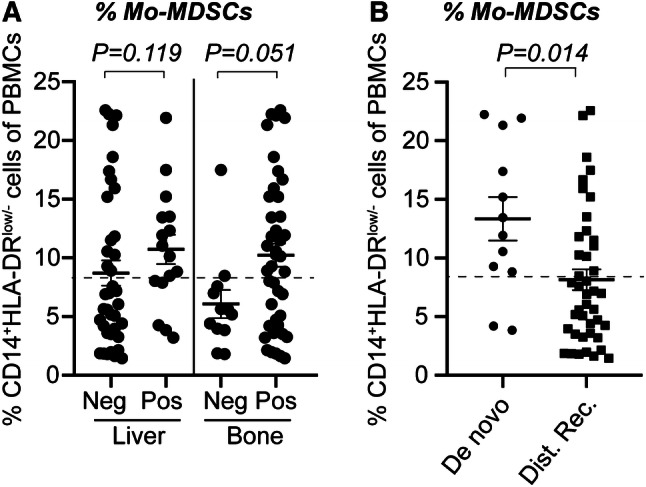
Percentages of Mo-MDSCs in patients divided by clinicopathological features. CD14^+^HLA-DR^low/−^ Mo-MDSCs in peripheral blood was assessed by flow cytometry and stratified according to clinicopathological features (see Table 1). Median percentage of Mo-MDSCs as divided by (**a**) metastatic site or (**b**) according to de novo or distant recurrent MBC. Error bars; SEM. Exact *P* values, by Mann–Whitney Wilcoxon, are indicated

In order to determine the clinical significance of Mo-MDSCs, clinicopathological features were compared in patients with normal *versus* high levels of Mo-MDSCs (Table 1). There was no significant difference between patients with normal- and high levels of Mo-MDSCs regarding age, performance status (Eastern Cooperative Oncology Group, ECOG), tumor type, size or histologic grade (Nottingham histological grade, NHG; Table 1). Significantly higher frequency of ER-negative primary tumors was seen among the patients with high levels of Mo-MDSCs compared to patients with normal levels (41% and 12% of patients, respectively; Table 1). Furthermore, significantly more liver metastases were seen in the group of patients with high levels of Mo-MDSCs compared to the group with normal levels of Mo-MDSCs (42% and 18% of patients, respectively; Table 1). Similarly, more patients with high levels of Mo-MDSCs had bone metastases compared to patients with normal Mo-MDSC levels (92% and 64%, respectively; Table 1 and Fig. 2a). Tendencies were also seen in correlations between high levels of Mo-MDSCs and metastatic burden (≥ 3 metastatic sites), number of circulating tumor cells (CTCs), and progression at three months’ radiology evaluation, where patients with high levels of Mo-MDSCs tended to have more metastatic sites, higher CTC levels, and more patients had progression at first evaluation (Table 1).

### High level of Mo-MDSCs is associated with de novo MBC

Interestingly, patients with de novo MBC were overrepresented in the group with high levels of Mo-MDSCs (Table 1). More than 80% of patients with de novo MBC had high Mo-MDSC levels, as compared with 38% of patients with distant recurrent MBC (*P* = 0.006; Tables 1, 2; Fig. 2b**)**. This dramatic difference urged us to clarify the role of Mo-MDSCs in the de novo MBC patients versus the distant recurrent MBC patients. No differences were seen between the two patient groups in terms of age, performance status, and several other important clinicopathological variables (Tables 1, 2). In the group with de novo MBC, all ten patients with high levels of Mo-MDSCs had bone metastasis (Table 2). The group with distant recurrent MBC patients with high levels of Mo-MDSCs tended to have more liver metastasis than patients with low Mo-MDSCs (*P* = 0.07; Table 2). Due to few patients in the de novo and distant recurrent MBC groups, differences were not statistically significant.

**Table 2:** Clinicopathological variables in de novo MBC *versus* distant recurrent MBC, stratified by levels of Mo-MDSCs

Clinicopathological variable	De novo MBC^a^		Distant recurrent MBC^b^		*P* value^c^
	Normal levels *n* = 2 (%)	High levels *n* = 10 (%)	Normal levels *n* = 26 (%)	High levels *n* = 16 (%)	
Age (years)					
< 65	1 (50)	7 (70)	12 (46)	7 (44)	1.0
≥ 65	1 (50)	3 (30)	14 (54)	9 (56)	
Baseline^d^ ECOG^e^					
0	1 (50)	6 (60)	19 (73)	9 (60)	0.70
1	0 (0)	0 (0)	3 (12)	3 (20)	
2	1 (50)	4 (40)	4 (15)	3 (20)	
Unknown	0	0	0	1	
Tumor type					
Ductal	2 (100)	8 (89)	20 (80)	10 (63)	0.31
Lobular	0 (0)	1 (11)	3 (12)	5 (31)	
Other	0 (0)	0 (0)	2 (8)	1 (6)	
Unknown	0	1	1	0	
PT NHG					
I	–	–	3 (13)	1 (8)	0.88
II	–	–	14 (58)	9 (69)	
III	–	–	7 (29)	3 (23)	
Unknown			4	13	
PT tumor size					
T1	0 (0)	0 (0)	13 (50)	7 (46)	0.30
T2	0 (0)	2 (29)	8 (31)	4 (27)	
T3	0 (0)	1 (14)	2 (8)	4 (27)	
T4	0 (0)	4 (57)	3 (11)	0 (0)	
Unknown	2	3	0	1	
PT node status					
Negative	0 (0)	0 (0)	11 (42)	4 (25)	0.33
Positive	0 (0)	4 (100)	15 (58)	12 (75)	
Unknown	2	6	0	0	
PT hormone receptor status					
ER-negative	0 (0)	4 (44)	3 (12)	5 (38)	0.09
ER-positive	1 (100)	5 (56)	22 (88)	8 (62)	
Unknown	1	1	1	3	
PR-negative	0 (0)	4 (44)	8 (32)	7 (58)	0.16
PR-positive	1 (100)	5 (56)	17 (68)	5 (42)	
Unknown	1	1	1	4	
PT HER2 status					
HER2-negative	0 (0)	6 (67)	18 (90)	8 (89)	1.0
HER2-positive	1 (100)	3 (33)	2 (10)	1 (11)	
Unknown	1	1	6	7	
MET hormone receptor status					
ER-negative	1 (50)	2 (33)	1 (4)	3 (19)	0.29
ER-positive	1 (50)	4 (67)	22 (96)	13 (81)	
Unknown	0	4	3	0	
PR-negative	1 (50)	3 (50)	12 (55)	11 (69)	0.51
PR-positive	1 (50)	3 (50)	10 (45)	5 (31)	
Unknown	0	4	4	0	
MET HER2 status					
HER2-negative	1 (50)	6 (100)	18 (86)	14 (93)	0.63
HER2-positive	1 (50)	0 (0)	3 (14)	1 (7)	
Unknown	0	4	5	1	
Metastatic sites, *n*					
< 3	1 (50)	4 (40)	19 (73)	9 (56)	0.32
≥ 3	1 (50)	6 (60)	7 (27)	7 (44)	
Metastatic sites, localization					
Lymph node					
Negative	0 (0)	2 (20)	18 (69)	11 (69)	1
Positive	2 (100)	8 (80)	8 (31)	5 (31)	
Lung					
Negative	1 (50)	7 (70)	15 (58)	10 (62)	1
Positive	1 (50)	3 (30)	11 (42)	6 (38)	
Liver					
Negative	1 (50)	6 (60)	22 (85)	9 (56)	0.07
Positive	1 (50)	4 (40)	4 (15)	7 (44)	
Bone					
Negative	1 (50)	0 (0)	9 (35)	2 (13)	0.16
Positive	1 (50)	10 (100)	17 (65)	14 (87)	
Visceral^f^					
Non-visceral	0 (0)	4 (40)	11 (42)	5 (31)	0.53
Visceral	2 (100)	6 (60)	15 (58)	11 (69)	
Bone only					
Not bone-only	2 (100)	9 (90)	20 (77)	12 (75)	1
Bone-only	0 (0)	1 (10)	6 (23)	4 (25)	
Progression at 3 months’ evaluation					
Non-progression	1 (100)	7 (88)	22 (92)	9 (64)	0.08
Progression	0 (0)	1 (12)	2 (8)	5 (36)	
Unknown	1	2	2	2	
CTC at baseline^d^					
< 5	1 (100)	4 (40)	15 (58)	5 (31)	0.12
≥ 5	0 (0)	6 (60)	11 (42)	11 (69)	
Unknown	1	0	0	0	

Number of patients and percentage (%) distribution of patients indicated for each variable

*MBC* metastatic breast cancer, *Mo-MDSCs* monocytic myeloid-derived suppressor cells, *ECOG* Eastern Cooperative Oncology Group, *PT* primary tumor, *ER* estrogen receptor, *PR* progesterone receptor, *HER2* human epidermal growth factor receptor 2, *MET* metastasis, *CTC* circulating tumor cells

^a^De novo MBC defined as MBC at initial breast cancer diagnosis, *n* too small for statistical analysis and no *P* values are listed

^b^Distant recurrent MBC defined as MBC diagnosis after > 0 years after primary diagnosis

^c^Statistics by Fisher’s exact test. Significance level defined as *P* < 0.05 (bold)

^d^Baseline defined as a time point before starting first line systemic MBC treatment

^e^ECOG denotes the performance status used in clinical practice in Sweden

^f^Visceral metastasis is defined as lung, liver, brain, peritoneal, and/or pleural involvement

### Associations of circulating Mo-MDSCs and outcome

**Fig. 3:**
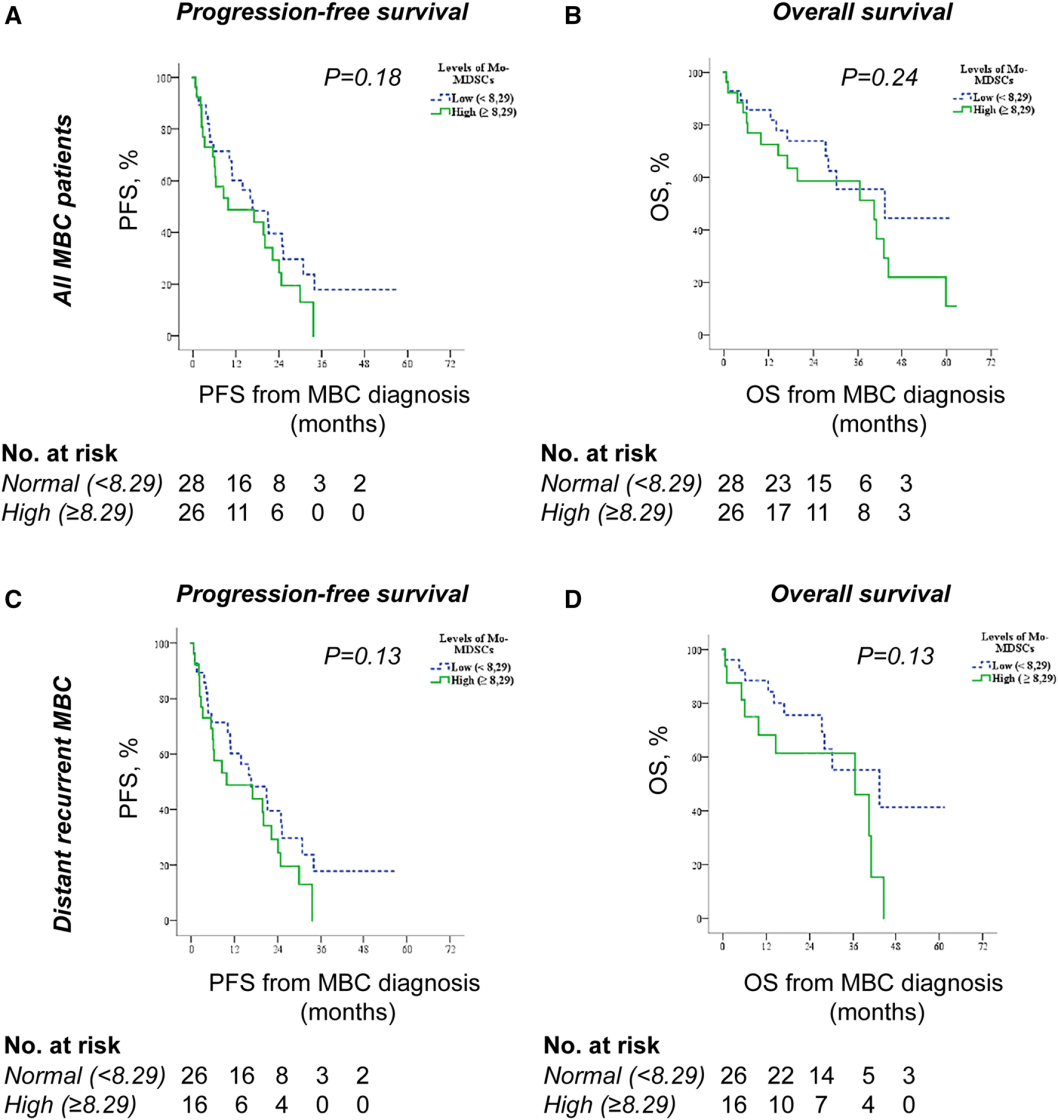
Mo-MDSC levels and associations with survival. Kaplan–Meier curves of progression-free survival (PFS) and overall survival (OS) according to Mo-MDSC levels in all MBC patients (*n* = 54, **a–b**) or in MBC patients with distant recurrence (*n* = 42, **c–d**). Statistics by Log-rank test

To evaluate the prognostic impact of Mo-MDSCs in all patients with MBC, we used Kaplan–Meier curves to compare progression-free survival (PFS) and overall survival (OS) in patients with normal and high Mo-MDSC levels. Patients with normal levels of Mo-MDSCs tended to have improved PFS compared to patients with high levels of Mo-MDSCs (median PFS; 16.6 months 95% CI 5.8–27.5 and 9.9 months 95% CI 0–25.1, respectively, *P* = 0.18; Fig. 3a). Similarly, OS tended to be better for patients with normal compared to high Mo-MDSC levels (median OS; 43.2 months 95% CI 12.3–74.1 and 40.3 months 95% CI 7.9–72.8, respectively, *P* = 0.24; Fig. 3b). This was specific for Mo-MDSCs as the levels of monocytes (all CD14^+^ cells, divided by median value 12.0% of PBMCs) did not relate to either PFS (Supplementary Fig. 2a) or OS (Supplementary Fig. 2b). Neither did the monocyte-levels (all CD14^+^ cells) correlate with any of the described clinicopathological parameters (data not shown).

As the role of Mo-MDSCs could be different in patients with de novo and distant recurrent MBC, we also assessed the impact of Mo-MDSCs in patients with distant recurrent MBC only. Similar tendencies regarding PFS (Fig. 3c) and OS (Fig. 3d) were observed. Median PFS of patients with distant recurrent MBC displaying normal and high Mo-MDSC levels were 20.9 months (95% CI 13.5–28.3) and 8.5 months (95% CI 2.0–15.1), respectively (*P* = 0.13; Fig. 3C), and median OS of patients with distant recurrent MBC displaying normal and high Mo-MDSC levels were 43.2 months (95% CI 15.5–71.0) and 36.5 months (95% CI 9.8–63.1), respectively (*P* = 0.13, Fig. 3d). This is in accordance with the correlation of high levels of Mo-MDSCs and disease progression at first radiology evaluation after 3 months of treatment (*P* = 0.08; Table 2) in this patient group.

**Fig. 4:**
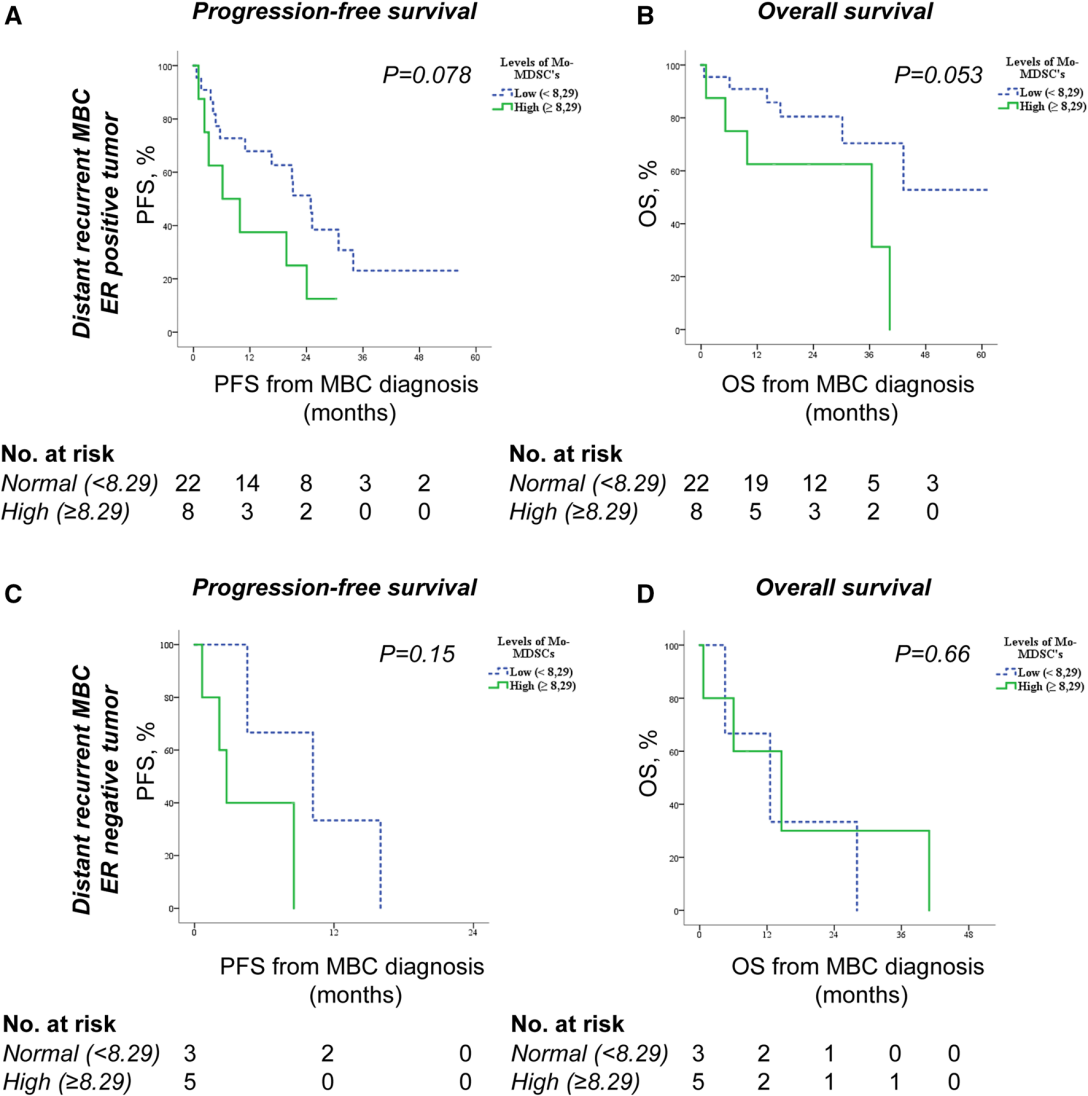
Mo-MDSC levels and associations with survival in distant recurrent MBC patients stratified for tumor ER status. Kaplan–Meier curves of progression-free survival (PFS) and overall survival (OS) in relation to Mo-MDSC levels in MBC patients with ER-positive primary tumors (*n* = 30, **a–b**) or in MBC patients with ER-negative primary tumors (*n* = 8, **c–d**). Statistics by Log-rank test

Finally, in subgroup analysis of patients with distant recurrent MBC, we found that high Mo-MDSC levels are more strongly associated with worse PFS and OS in patients with ER-positive tumors (Fig. 4a–b) than ER-negative tumors (Fig. 4c–d). In patients with ER-positive primary tumors and high Mo-MDSC levels, median PFS was 6.2 months (95% CI 0–15.3) compared to 24.9 months (95% CI 17.5–32.3) for patients with low Mo-MDSC levels *(P* = 0.078; Fig. 4a). For OS similar associations were seen with worse survival in patients with high Mo-MDSC levels (*P* = 0.053; Fig. 4b). Also, when analyzing Mo-MDSC levels and possible correlations to survival in all patients (de novo and distant recurrent MBC) stratified for tumor ER status, there was a trend towards impaired OS in patients with high Mo-MDSC levels in the ER-positive tumor group (*P* = 0.06), whereas in the group of patients with ER-negative tumors there was no difference in OS in relation to Mo-MDSC levels (Supplementary Fig. 3).

## Discussion

In the present study we evaluated the role of systemic Mo-MDSCs in patients with newly diagnosed MBC. A high level of Mo-MDSCs was significantly correlated with ER-negative primary tumors, as well as with liver- and bone metastases, which is in accordance with our previous study, comprising the first 23 patients enrolled in this study [28]. This is also in line with a previous study proposing that circulating G-MDSCs may be more predominant in triple-negative breast cancer compared to other breast cancer subtypes [21]. ER-negative breast cancer has been associated with a stronger inflammatory response, where more inflammatory cytokines (e.g., IL-8 and IL-6) have been observed in tumors of patients with ER-negative breast cancer or in ER-negative breast cancer cell-lines [33, 34]. Pro-inflammatory stimulus is one important component of MDSC generation and accumulation [12]. The higher inflammatory activity associated with ER-negative breast cancer may consequently promote the accumulation of Mo-MDSCs.

Interestingly, the vast majority of patients with de novo MBC displayed high levels of Mo-MDSCs. De novo MBC differs from other breast cancer forms in terms of both characteristics and treatment strategies. Compared to early breast cancer, de novo MBC is a disease with systemic involvement already at first diagnosis. Though, unlike patients with distant recurrent MBC, where the primary tumor had been resected at initial diagnosis, the primary tumor is still present in the subgroup of patients with de novo MBC. This implies that the primary tumor might impact the levels of Mo-MDSCs and that different biological mechanisms may be at play in patients with de novo MBC and distant recurrent MBC. Indeed, the levels of MDSCs decrease after removing the primary tumor in breast cancer mouse models and patients with MBC [19, 35]. Studies have shown that cytokines such as GM-CSF, produced by tumor cells, may induce expansion of MDSCs [12, 13]. It is also interesting to note that patients with early, localized, breast cancer generally display a modest enrichment in circulating Mo-MDSCs as compared to patients with locoregional recurrence or MBC [28]. The enrichment of Mo-MDSCs may thus be related to the metastatic or immunoregulatory switch during the transition to a more systemic disease [36]. This is supported by a study proposing that Mo-MDSCs themselves may induce a pro-metastatic phenotype of 4T1 murine breast tumor cells [37].

The strong relationship between Mo-MDSCs and liver- and bone metastases, as well as the observed associations with more CTCs, more metastatic sites, disease progression, and shorter progression-free and overall survival, indicate that Mo-MDSCs are related to a more aggressive metastatic disease and worse prognosis. This association was especially apparent in patients with distant recurrent MBC and is in line with previous studies where MDSCs in general are associated with clinical stage, metastatic burden and poor overall survival in breast cancer patients [17, 19]. The potential correlation between Mo-MDSCs and CTCs was hitherto unexplored in breast cancer patients, although it has been proposed that MDSCs (CD11b^+^CD33^+^Lin^−^HLA-DR^−^ cells) may correlate with CTCs [16]. Interestingly, CTCs have been suggested to preferably cluster together with leukocytes and that these clusters might be associated with worse prognosis in breast cancer patients [38]. The specific leukocyte population(s) was until recently uncharacterized. However, MDSC-like pro-tumor N2 neutrophils were recently shown to support the metastatic potential of CTCs in patients with metastatic breast cancer [39]. Whether also Mo-MDSCs, specifically, may play a role will be of interest to delineate in the future.

Tumor metastasis is a complex process and the outcome depends on both tumor properties and the host’s response, accounting for the fact that only 0.01% of the malignant cells are estimated to succeed in establishing micro-metastases [40]. The process of establishing new metastatic sites is not fully understood, but has been proposed to involve formation of pre-metastatic niches. Intriguingly, several MDSC-associated mediators (such as S100A8/A9, MMP9 and IL-1β) have been identified as important in establishment of pre-metastatic niches [40, 41]. Accordingly, mouse models of malignant melanoma have shown that both Mo-MDSCs and G-MDSCs are found in pre-metastatic sites weeks before the actual metastasis could be detected [41]. In fact, Mo-MDSC-derived cytokines have been proposed to facilitate adhesion of CTCs to the endothelium in the pre-metastatic organ [41]. How and why Mo-MDSCs would localize to pre-metastatic niches is not fully characterized, nor is it known whether this also occurs in human patients [42]. It is tempting to speculate that correlation between high levels of circulating Mo-MDSCs and specific metastatic sites, predominantly liver and bone, may be related to establishment of pre-metastatic niches by MDSCs [43]. This would also be in line with our hypothesis that enrichment of Mo-MDSCs is related to the metastatic and immunoregulatory switch in these patients. Further we show that high Mo-MDSC levels may be associated with impaired survival. This seems to be particularly relevant in patients with ER-positive MBC, a patient group with generally better prognosis compared to ER-negative MBC, implying that Mo-MDSC levels could be a prognostic factor in this subgroup of patients representing a more aggressive disease. Thus, although the inflammatory environment in ER-negative tumors promotes accumulation of Mo-MDSCs to a greater extent, the impact of Mo-MDSCs on survival may be limited due to the overall worse prognosis in this subgroup of patients.

Immune therapies are currently revolutionizing the field of oncology and check point inhibitors are being implemented as systemic therapies for different tumor types including breast cancer [44, 45]. For MBC the PD-L1 inhibitor atezolizumab was recently shown to improve PFS and OS in patients with metastatic TNBC [46]. Mo-MDSCs are cells with capability to modulate the immune response in MBC patients and could hence be a potential target for immune therapies in the future. Our study is limited by the sample size, and some analyses thus fail to reach statistical significance. Despite this, strong trends are observed shedding light on the clinical impact of systemic Mo-MDSCs in breast cancer patients. An extended knowledge of the systemic immune response is, therefore, extremely relevant in understanding what patients will benefit most from immune therapies.

### Electronic supplementary material

Below is the link to the electronic supplementary material.

Supplementary file1 (PDF 3851 kb)

## Abbreviations

AUC: Area under the curve
CTC: Circulating tumor cells
ECOG: Eastern Cooperative Oncology Group
ER: Estrogen receptor
G-MDSCs: Granulocytic myeloid-derived suppressor cells
HER2: Human epidermal growth factor receptor 2
MBC: Metastatic breast cancer
MDSCs: Myeloid-derived suppressor cells
MET: Metastasis
MFI: Metastasis-free interval
Mo-MDSCs: Monocytic myeloid-derived suppressor cells
NHG: Nottingham histological grade
OS: Overall survival
PBMCs: Peripheral blood mononuclear cells
PFS: Progression-free survival
PR: Progesterone receptor
PT: Primary tumor
ROC: Receiver operating characteristic
