# Supplementary material for: Clinical relevance of systemic monocytic-MDSCs in patients with metastatic breast cancer
Source: Cancer Immunol Immunother. 2020 Jan 10;69(3):435–48. doi: 10.1007/s00262-019-02472-z (PMC7044142; doi:10.1007/s00262-019-02472-z)
Supplement: Supplementary file 1 — Supplementary file1 (PDF 3851 kb) [file 262_2019_2472_MOESM1_ESM.pdf]

Supplementary Figure 1

A

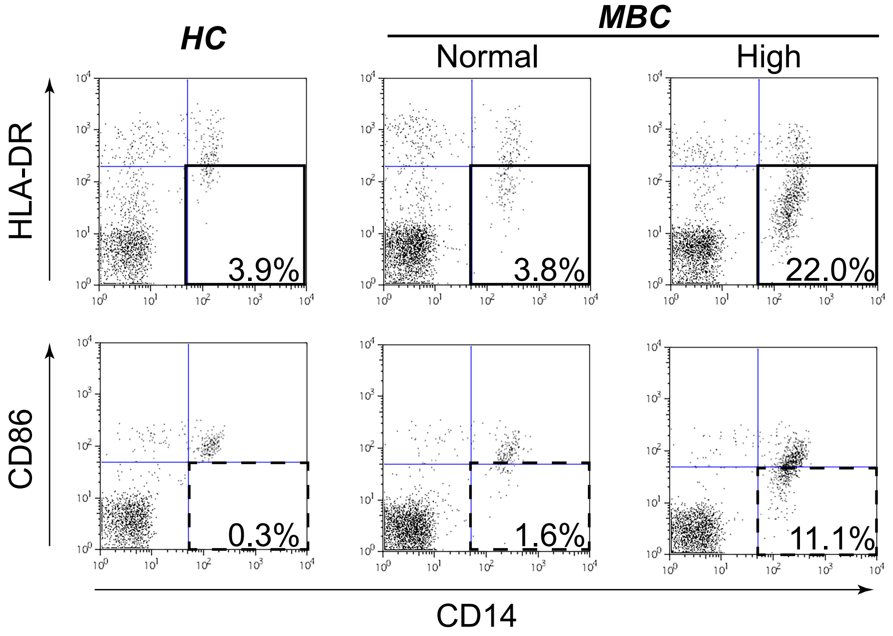

B

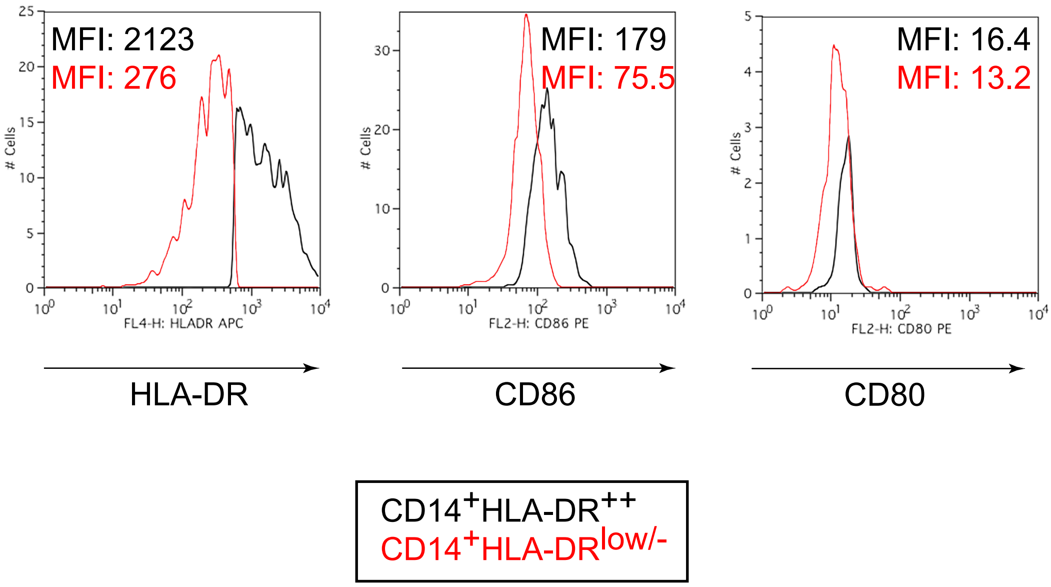

Supplementary Figure 1. Flow cytometric analyzes of CD14$^+$HLA-DR$^{low/-}$ and CD14$^+$HLA-DR^{++} cells. (**A**) Representative dot plots of CD14, HLA-DR and co-receptor CD86, gated on all viable PBMCs. Mo-MDSCs were defined as CD14$^+$HLA-DR$^{low/-}$ cells (black box). All CD14$^+$HLA-DR$^{low/-}$ cells had lower expression of CD86 (dashed box) as compared to CD14$^+$HLA-DR^{++} cells. (**B**) Representative histograms of mean fluorescence intensity (MFI) for HLA-DR, CD86 and CD80 in CD14$^+$HLA-DR^{++} (black) and CD14$^+$HLA-DR$^{low/-}$ (red) cells.

Supplementary Figure 2

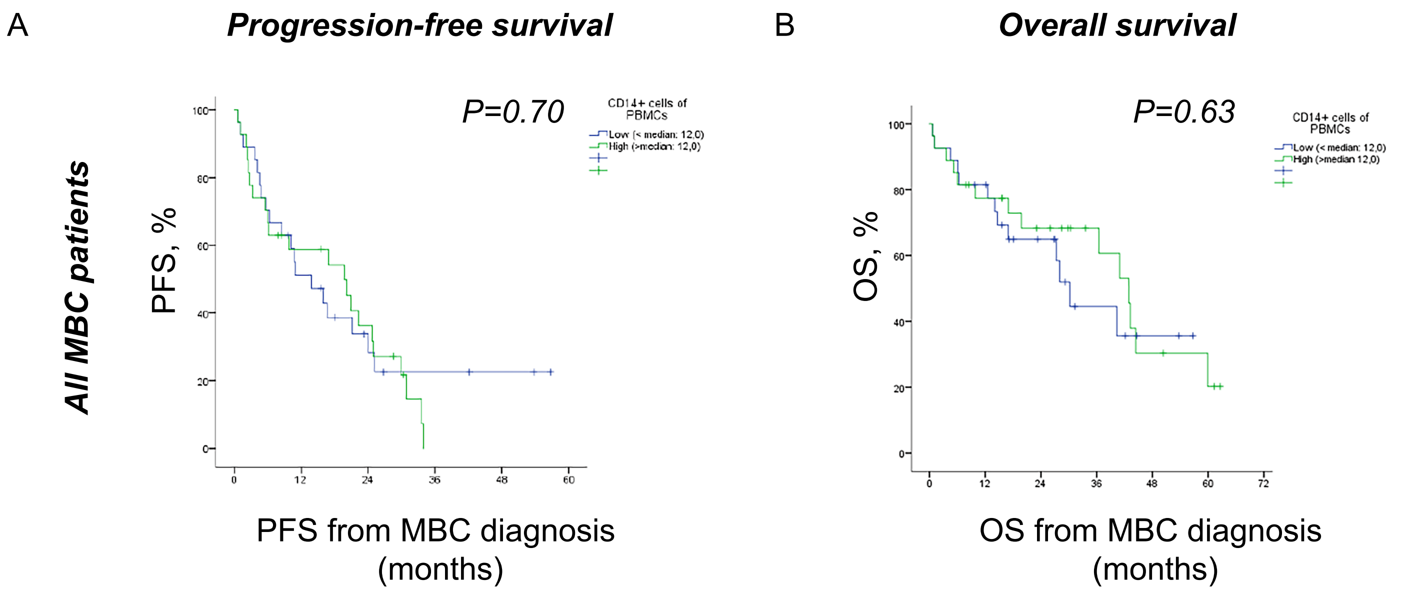

Supplementary Figure 2. Monocyte levels and associations to survival. (**A-B**) Kaplan-Meier curves of progression-free survival (PFS; A) and overall survival (OS; B) according to the monocyte levels (all $CD14^{+}$ cells), divided by median level of monocytes, 12.0 % of PBMCs, in all MBC patients (*n*=54). Median PFS in low and high group was 13.8 months (95% CI 5.7-21.9) and 19.7 months (95% CI 7.7-31.8), respectively. Median OS in low and high group was 30.2 months (95% CI 25.4-34.9) and 42.9 months (95% CI 35.7-50.1), respectively. Statistics by Log-rank test.

Supplementary Figure 3

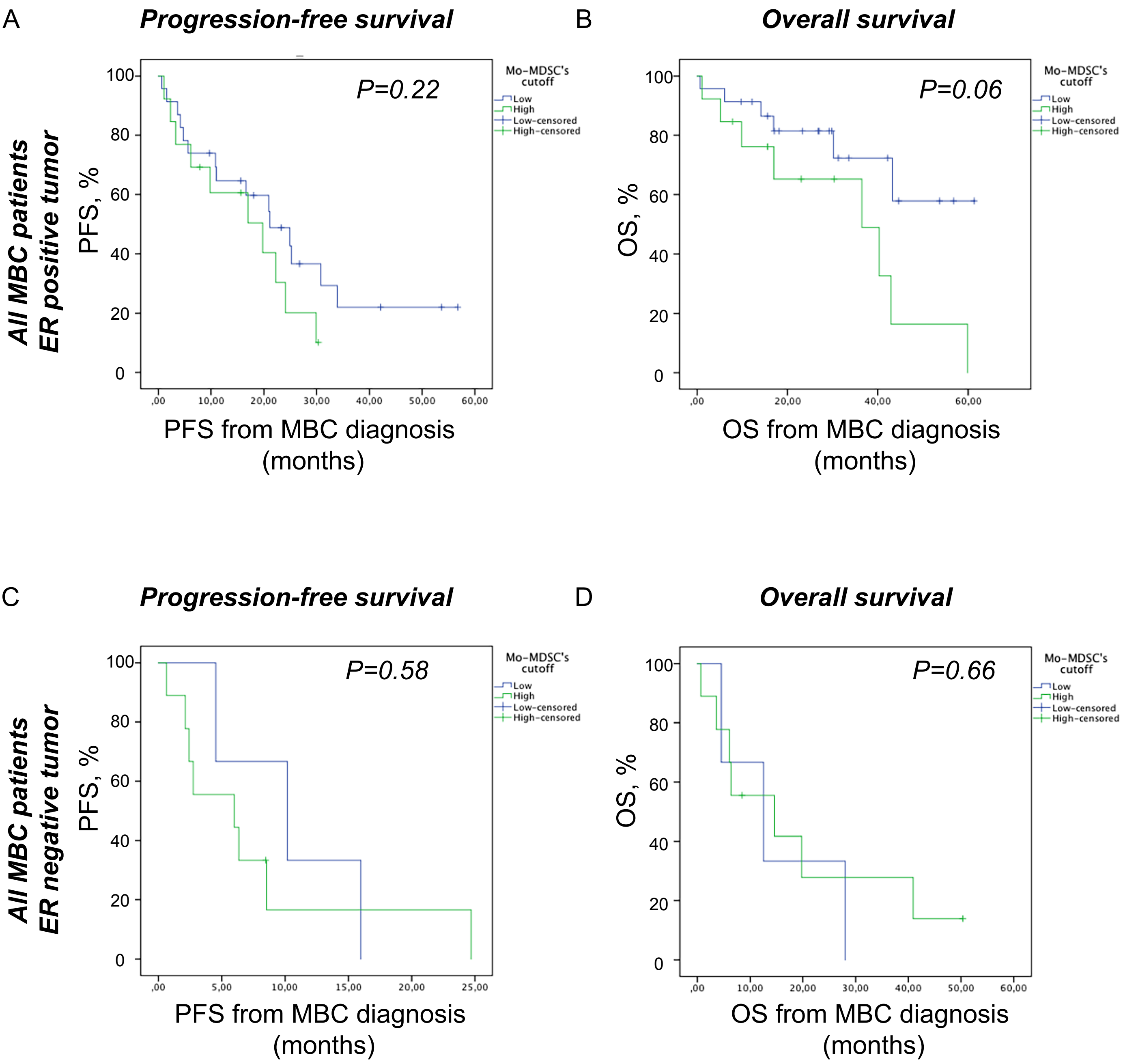

Supplementary Figure 3. Mo-MDSC levels and associations to survival in all MBC patients stratified for tumor ER status. Kaplan Meier curves of progression-free (PFS) and overall survival (OS) in relation to Mo-MDSC levels in MBC patients with ER positive (*n*=36, **A-B**) versus ER negative (*n*=12, **C-D**) disease. Statistics by log rank test.

Supplementary Table 1. Table illustrating previous adjuvant therapy (chemotherapy and endocrine therapy) for early breast cancer in 42 patients diagnosed with distant recurrent MBC. [a] Statistics by Pearson Chi Square test. [b] Statistics by Fisher's exact test.

Type of therapy		***Normal Mo-MDSCs*** *n*=26	***High Mo-MDSCs*** *n*=16	***P value***
**Chemotherapy**	Yes	11	10	0.20[a]
	No	15	6	
	Unknown	0	0	
**Endocrine therapy**	Yes	22	12	0.45[b]
	No	4	4	
	Unknown	0	0	
**Endocrine therapy at MBC diagnosis**	Yes	10	3	0.30[b]
	No	14	12	
	Unknown	2	1	
